# Comparing the Prevalence of Chronic Pain After Sternotomy in Patients Undergoing Coronary Artery Bypass Grafting Using The Internal Mammary Artery and Other Open Heart Surgeries

**DOI:** 10.5812/aapm.17969

**Published:** 2014-06-21

**Authors:** Hamid Kamalipour, Ali Vafaei, Asef Parviz Kazemi, Saeed Khademi

**Affiliations:** 1 Laparoscopy Research Center, Shiraz University of Medical Sciences, Shiraz, Iran; 2 Anesthesiology and Critical Care Research Center, Shiraz University of Medical Sciences, Shiraz, Iran

**Keywords:** Chronic Pain, Cardiac Surgery, Sternotomy, Internal Mammary Artery

## Abstract

**Background:**

The prevalence of chronic postoperative pain after cardiac surgery has been reported from 17% to 56%.

**Objectives:**

We aimed to compare the prevalence of postoperative pain between patients who had undergone CABG using the internal mammary artery (IMA) and those who had undergone other cardiac surgeries including CABG using the saphenous vein or cardiac valvular surgeries.

**Patients and Methods:**

In this cohort study, medical records of 188 patients were evaluated and divided into two equal groups (94 in each group); patients who had undergone CABG using the IMA (IMA group) and those who had undergone other cardiac surgeries using the saphenous vein or other cardiac valvular surgeries (non-IMA group). The patients' data were recorded in a self-structured questionnaire and then phone interviews were performed 3 months after the operations regarding the rate of postoperative pain. The severity of chronic pain was rated based on the numerical rating pain scale.

**Results:**

The two groups differed significantly regarding the prevalence of pain (P = 0.023). In the IMA group, 83 (88.3%) patients experienced pain lasting for more than three months compared to 71 (75.5%) patients in non-IMA group. The two groups differed significantly with respect to the severity of chronic pain after cardiac surgery via sternotomy (P = 0.001). The groups did not differ significantly regarding the effects of chronic pain on their sleep, referral to a physician, and drug consumption to alleviate their pain. The IMA group experienced more complications at work and during their occupational activity.

**Conclusions:**

The rate and severity of chronic pain after cardiac surgery via sternotomy was higher in patients undergoing CABG with separation of IMA for revascularization.

## 1. Background

According to the International Association for the Study of Pain (IASP), chronic pain is a pain without any determined biological rate which persists after the normal rehabilitation period of a tissue for about three months (1). Despite fundamental developments in understanding pain mechanisms and treatments, postoperative chronic pain is still in an undesirable state (2) the importance of this issue was determined for the first time in 1992 in a study on patients referring to a pain clinic in Scotland. In this study, 20% of the patients stated that surgery was the leading factor affecting their pain as well as the only cause of pain in 50% of the cases (2). Studies show that inadequate pain relief after surgeries can have negative physiological and psychological consequences, delay recovery, interfere with normal organ function, and ultimately lead to increased mortality and morbidity rates (2, 3).

Cardiac surgery is a potentially stressful process for patients since they are afraid of the final outcome of surgery, and believe that if they survive, sustained postoperative pain would affect and limit their routine life (4). Similar to any invasive technique used in cardiac surgery, open heart surgery with the sternotomy approach is painful. Trauma and distributed tissue damage increase during cardiac surgery leading to the release of inflammatory mediators from dead or damaged cells which play a role in creating pain (4). Based on previous studies chronic chest pain after cardiac surgery via sternotomy is a serious problem for 17-56% of patients (5). Moreover, patients undergoing coronary artery bypass grafting (CABG) by the separation of the internal mammary artery (IMA) express more severe pain after surgery (6). During cardiac surgery with the separation of the IMA, soft tissues and nerves (intercostal and brachial nerve network) are damaged. This damage could be caused by the asymmetrical and prolonged traction of the opened sternal halves leading to higher brachial network damage and consequently chronic and sustained pain after surgery (7).

## 2. Objectives

Considering the importance of this issue, we aimed to compare the prevalence of postoperative pain between the two groups of patients; patients who had undergone CABG using the IMA (IMA group) and those who had undergone other cardiac surgeries including CABG using the saphenous vein or cardiac valvular surgeries (non-IMA group) to understand, follow, and treat factors leading to chronic postoperative pain.

## 3. Patients and Methods

In this cohort study, medical records of 188 patients referred to Nemazee and Shahid Faghihi Hospitals, Shiraz, Southern Iran during 2010-2012 were evaluated and divided into two equal groups (94 in each group); patients who had undergone CABG using the IMA (IMA group) and those who had undergone other cardiac surgeries including CABG using the saphenous vein or cardiac valvular surgeries (non-IMA group). The patients were in classes II and III of the American Society of Anesthesiologists (ASA) physical status class. The sample size was calculated using previous studies (P1 = 15%, P2 = 35%, α = 5%, 1-B = 90%) as 94 patients in each group. The patients' data were recorded in a self-structured questionnaire and then phone interviews were performed regarding the rate of postoperative pain. The interviews were performed by an anesthesiology resident three months after the operations. The severity of chronic pain was rated based on the numerical rating pain scale (NRS) on a scale of 0-10 (0 = no pain, 1-3 = mild, 4-6 = moderate, and 7-10 = severe pain). General anesthesia was induced using the standard narcotic-based protocol for all the patients: midazolam, fentanyl, morphine, sodium thiopental for anesthesia induction and pancuronium for paralysis and maintained with isoflurane and fentanyl. Otherwise the patient was excluded. The sternum was incised using classic midline sternotomy in all patients and hypothermia during operation was in the range of 34-36 °C. Pain management was similar for all patients (diclofenac suppository and/or intravenous pethidine) in ICU. We included 25-65 year-old patients who had undergone cardiac surgery via sternotomy at least three months prior to the study, with an ejection fraction of more than 40%. The exclusion criteria were as follows: redosurgery, kidney and liver failure, malignant neoplasms, alcoholism, chronic psychological problems needing medication, ICU stay of more than 5 days due to respiratory complications and wound infection, addiction, and death. Patients with incomplete records or those unwilling to cooperate or were unreachable phone interviews were also excluded and replaced. Data was analyzed using SPSS software, version 19 (SPSS Inc., Chicago, Illinois). Chi-square, T-tests and Pearson’s correlation coefficient were used where necessary.

## 4. Results

The mean ± SD ages of the participants in the IMA and non-IMA groups were 54.5 ± 7.3 and 46.28 ± 14.2 years (range: 25-65 years), respectively. The patients’ demographic variables are summarized in Table 1. The operations length ranged from 3 to 5 hours. Based on Pearson’s correlation coefficient, no significant difference was found between age and severity of pain in the both groups. The postoperative ICU stay was similar in both groups and ranged from 2-5 days with a maximum of 3 days. In our study, 140 (74.5%) patients did not have diabetes mellitus and 48 (25.5%) had this disease. We found a significant difference between the mean ± SD pain scores in patients with and without diabetes mellitus (P = 0.006). Moreover, we only found a significant difference between the mean pain scores of patients with and without diabetes in the IMA group (P = 0.01) but not in the non-IMA group (P = 0.8). With respect to sex, no significant difference was found between the mean pain scores of men and women in the IMA (P = 0.06) and non-IMA (P = 0.58) groups. Table 2 shows the mean ± SD pain scores in both groups with respect to some variables. The two groups differed significantly regarding the prevalence of pain (P = 0.023). In the IMA group, 83 (88.3%) patients experienced pain lasting for more than three months compared to 71 (75.5%) patients in the non-IMA group. The IMA group experienced more pain in all different positions (rest, standing, bending forward, mounting stairs, deep breathing or coughing, during housework) compared with the non-IMA group (Table 3). In both groups most patients had moderate chronic pain after cardiac surgery via sternotomy (66% in the IMA group vs. 41.5% in the non-IMA group). Severe pain was experienced in 9 (9.6%) and 3 (3.2%) patients in the IMA and non-IMA groups, respectively. The two groups differed significantly regarding the severity of chronic pain after cardiac surgery via sternotomy (P = 0.001). The two groups did not differ significantly regarding the effects of chronic pain on their sleep, referral to a physician, and drug consumption to alleviate their pain. However, the IMA group experienced more complications at work and during their occupational activity compared with the non-IMA group (p = 0.001, Table 4). Moreover, pain in sites other than the site of operation (shoulders, neck, and upper extremities) was more prevalent in the IMA group compared with the non-IMA group (P < 0.001, Table 5). We also found a significant difference between the two groups regarding localized pain at surgical site or being distributed throughout the chest (P < 0.001). In the IMA group, 30 (31.9%) patients had pain at the site of surgery, while 56 (59.6%) had distributed pain throughout the chest. The corresponding figures for the non-IMA group were 48 (51.1%) and 23 (24.5%), respectively.

**Table 1.** Comparison of Frequency (%) and/or Mean ± SD of Some Related Variables in The IMA and Non-IMA Groups

Variable	IMA Group	Non-IMA Group
**Ejection Fraction 50-60%**	53 (56.4)	54 (57.4)
**Diabetes Mellitus**		
Yes	35 (37.2)	13 (13.8)
No	59 (62.8)	81 (86.2)
**Sex**		
Male	55 (58.5)	41 (43.6)
Female	39 (41.5)	53 (56.4)
**History of Smoking**		
Yes	27 (28.7)	15 (16)
No	67 (71.3)	79 (84)

**Table 2.** Mean ± SD Pain Scores in the IMA And Non-IMA Groups Regarding Some Variables

Variable	IMA Group	Non-IMA Group	Mean ± SD	P Value
**Diabetes Mellitus**				0.006
Yes	5.02 ± 1.48	3 ± 1.68	4.74 ± 1.77	
No	4.05 ± 1.88	3.1 ± 2.2	3.53 ± 2.1	
**Sex**				0.41
Male (n = 96)	4.14 ± 2.09	3 ± 2.4	3.65 ± 2.29	
Female (n = 92)	4.79 ± 1.21	3.24 ± 1.91	3.9 ± 1.81	
**History of Smoking**				0.42
Yes (n = 42)	4.33 ± 2.27	3.4 ± 2.22	4 ± 2.27	
No (n = 146)	4.44 ± 1.59	3.08 ± 2.12	3.71 ± 2.01	

**Table 3.** Frequency (%) of Patients Experiencing Pain in Different Positions

Position in Which Pain is Intensified	IMA Group	Non-IMA Group	P Value
**Rest**	25 (26.6)	8 (8.5)	0.001
**Standing**	28 (29.8)	8 (8.5)	< 0.001
**Bending Forward**	71(75.5)	44 (46.8)	< 0.001
**Mounting Stairs**	80 (85.1)	66 (70.2)	0.014
**Deep Breathing or Coughing**	75 (79.8)	51 (54.3)	< 0.001
**During House Work**	81 (86.2)	64 (68.1)	< 0.003

**Table 4.** Comparison of the Effect of Chronic Pain on Different Variables Between the Two Groups (Frequency %)

Variable	P Value	Non-IMA Group	IMA Group
**Sleep Disorders**	0.1	10 (10.6)	18 (19.1)
**Referral to a Physician During the Past Three Months**	0.17	1 (1.1)	4 (4.3)
**Drug Consumption During the Past Two Months**	0.57	8 (8.5)	6 (6.4)
**Occupational Disorders**	0.001	16 (17)	42 (44.7)

**Table 5.** Comparing the Site of Pain in Parts Other Than the Surgical Site Between the Two Groups

Site	IMA Group, Frequency (%)	Non-IMA Group, Frequency (%)	P Value
**Shoulders**	67 (71.3)	44 (46.8)	0.001
**Neck**	58 (61.7)	24 (25.5)	< 0.001
**Upper Extremities**	42 (44.7)	16 (17)	< 0.001

## 5. Discussion

We found that chronic pain after cardiac surgery was more prevalent in the IMA group (88.3%) than the non-IMA group (75.5%). This percentage was considerably higher than other related studies, which could be attributed to different surgical techniques during sternotomy and separating the IMA, duration of operation, patient's position, and postoperative acute pain management. In previous studies, chronic postoperative pain after cardiac surgery through sternotomy has been reported as 30% (3) and 56% after CABG (8). Moreover, studies reported a 20.6% brachial damage during cardiac surgery using the IMA (9, 10). Factors that create or intensify chronic pain after cardiac surgery were assessed in the two study groups in 6 different positions. Postoperative pain was more prevalent in the IMA group in all six positions. In another study performed on patients who had undergone CABG, 72% of patients reported pain that interfered with their daily life; (8) while, in another study 39.1% of patients reported unbearable chronic pain after the operation (11). The severity of postoperative pain was evaluated using the NRS. The prevalence of severe chronic postoperative pain was 9.6% and 3.2% in the IMA and non-IMA groups, respectively. Moderate pain was observed in 66% and 41.5% of the patients in the IMA and non-IMA groups, respectively. The corresponding figures for mild postoperative pain in the IMA and non-IMA groups were 16% and 30.9%, respectively. In a study on postoperative chronic pain after cardiac surgery via sternotomy mild, moderate, and severe pain was reported as 14%, 1%, and 2% (6). We did not find a significant difference between the IMA and non-IMA groups regarding the rate of sleep disorder and amount of consumed medication to alleviate pain. In total, 44.7% and 17% of the patients in the IMA and non-IMA groups reported that their pain interfered with their occupation. Regarding the rate of pain in sites other than the site of operation, we found that the IMA group experienced pain in shoulders, neck, and upper extremities more than the non-IMA group. In another study on chronic pain after cardiac surgery via sternotomy, the rate of shoulder, neck and back pain was lower than our study (6). We found that localized chronic pain at the site of sternum wound was lower in the IMA group while distributed chronic pain in chest was higher in this group compared with the non-IMA group. In a previous study, researchers reported a prevalence of 29% for pain at surgical sites and a prevalence of 25% for pain at the sternotomy site (6). Considering the high prevalence of chronic pain in the IMA group (88%) and the increasing rate of cardiac surgeries using the IMA, it is necessary to follow patients before, during, and after the operation and present methods to minimize their pain experience. Several approaches have been mentioned to reduce peripheral nerve damage, especially to the brachial network, as a factor for creating chronic pain after the operation, as follows: (12) median sternotomy should be performed correctly, caudal placement of the retractor, preventing prolonged or asymmetrical traction of the opened sternal halves, using asymmetrical traction retractors with more caution (9), and the arms should be held up for separating the IMA to prevent traction to the brachial network (12-14). Recently, by presenting methods for the separation of the IMA with thoracoscopy as well as performing CABG using less invasive methods, high sternum traction is prevented (13). Moreover, establishing a service center for controlling acute pain to identify, follow, and treat acute postoperative pain as a factor for creating chronic pain is recommended. Considering the high prevalence of chronic postoperative pain after cardiac surgery via sternotomy in cases using the IMA, the timely and early identification of effective factors and the patients at risk, facilitates the treatment of chronic pain (13). Moreover, postoperative chronic chest pain should be described for high risk patients to increase their awareness (14). Our knowledge regarding the etiology, prevention, and treatment of chronic chest pain is limited and more studies should be performed in this regard (13).

This study had some limitations such as incomplete patients' records, lack of patient’s cooperation, and being unable to reach patients. In conclusion, the rate and severity of chronic pain after cardiac surgery via sternotomy was higher in the IMA group compared to the non-IMA group. It is necessary to present methods to reduce chronic pain in such patients (15, 16).
